# Variations in Dream Recall Frequency and Dream Theme Diversity by Age and Sex

**DOI:** 10.3389/fneur.2012.00106

**Published:** 2012-07-04

**Authors:** Tore Nielsen

**Affiliations:** ^1^Department of Psychiatry, Université de MontréalMontréal, QC, Canada; ^2^Dream and Nightmare Laboratory, Center for Advanced Research in Sleep Medicine, Hôpital du Sacré-Coeur de MontréalMontréal, QC, Canada

**Keywords:** dreaming, dream recall, dream diversity, sleep, aging, sex differences, epidemiology

## Abstract

We assessed dream recall frequency (DRF) and dream theme diversity (DTD) with an internet questionnaire among a cohort of 28,888 male and female participants aged 10–79 years in a cross-sectional design. DRF increased from adolescence (ages 10–19) to early adulthood (20–29) and then decreased again for the next 20 years. The nature of this decrease differed for males and females. For males, it began earlier (30–39), proceeded more gradually, and reached a nadir earlier (40–49) than it did for females. For females, it began later (40–49), dropped more abruptly, and reached nadir later (50–59). Marked sex differences were observed for age strata 10–19 through 40–49 and year-by-year analyses estimated the window for these differences to be more precisely from 14 to 44 years. DTD decreased linearly with age for both sexes up to 50–59 and then dropped even more sharply for 60–79. There was a sex difference favoring males on this measure but only for ages 10–19. Findings replicate, in a single sample, those from several previous studies showing an increase in DRF from adolescence to early adulthood, a subsequent decrease primarily in early and middle adulthood, and different patterns of age-related decrease in the two sexes. Age-related changes in sleep structure, such as decreasing %REM sleep which parallel the observed dream recall changes, might help explain these findings, but these sleep changes are much smaller and more gradual in nature. Changes in the phase and amplitude of circadian rhythms of REM propensity and generational differences in life experiences may also account for some part of the findings. That decreases in DTD parallel known age-related decreases in episodic and autobiographical memory may signify that this new diversity measure indexes an aspect of autobiographical memory that also influences dream recall.

## Introduction

The recallability, content, and organization of dreaming reflect influences from underlying circadian and ultradian fluctuations in cognitive activity (Wamsley et al., 2007; Nielsen, 2011). Age-related changes in dreaming might thus be expected to parallel changes seen in other domains of cognitive functioning, such as episodic or autobiographical memory (St-Laurent et al., 2011), or even to reveal age-related changes in cognitive activity that are unique to sleep (Pace-Schott and Spencer, 2011).

There is a relatively broad consensus, based on results from both survey and laboratory studies, that the capacity to recall dreams decreases with age (for reviews see Funkhouser et al., 1999; Guenole et al., 2010). One early study of 17- to 70-year-old college-educated participants (*n* = 295) found that dream recall frequency (DRF) was at its highest level (9.8 dreams/month) in the late teens, progressively lower at ages 30–39 (6.1/month), 40–49 (4.2/month), and 50–59 (3.7/month) and then somewhat higher again at ages 60–69 (4.5/month; Herman and Shows, 1983). A second measure of dream recall in this study, the number of separate dreams recallable in 5 min, produced a clear linear decrease with age. A subsequent, much larger, cross-sectional study of participants aged 17–92 years (*n* = 2328) found progressively lower DRF with increasing age, but only up to the age of 56, when no further decrease was apparent (Giambra et al., 1996). In contrast to these findings, however, a home diary study (Waterman, 1991) found no difference in dream recall or dream report length between middle-aged (45–60 years) and older (61–75 years) participants. This may be attributable to the lack of a younger comparison group in this study (see later section).

Findings from laboratory studies have been largely consistent with those of surveys. The first laboratory study of this question (Kahn et al., 1969) found a relatively low proportion of dream recall after awakenings from REM sleep among a 66- to 87-year-old group (45%) compared with that of a younger, 18–33 year old, group (87%) reported in a previous study (Kahn et al., 1962). A subsequent study (Fein et al., 1985) also reported lower dream recall from REM sleep awakenings for a 69- to 75-year-old group of women (71.4%) than for a younger comparison group of unspecified age (90.2%), as well as a trend for lower recall from Stage 2 sleep awakenings (47.2 vs. 60.3%). The length of reports did not differentiate the old and young groups however. A third study (Grenier et al., 2005) reported lower REM sleep dream recall in a group of 60- to 77-year-old women (81%) than in a comparison group of 18- to 35-year-old women (98%); the mean word count of the older women’s dreams was also lower than that of the younger women.

Several studies suggest that this reliable age-related decrease in DRF occurs abruptly at a young age rather than progressively over the lifespan. First, the survey study cited earlier (Herman and Shows, 1983) reported a sudden decrease in DRF between 17–20 year-olds and those aged 30–69. Similarly, Kahn et al. (1969) found that the biggest decrease in DRF occurred between 25 and 35 years of age, while Giambra et al. (1996) found the decrease to occur between ages 20 and 38.

Some studies have also reported a sex difference in DRF, with women reporting higher recall frequencies than men (Giambra et al., 1996). A meta-analysis (Schredl and Reinhard, 2008) confirms the presence of such a difference, indicating that it is small in magnitude, and especially small in children. Moreover, our previous longitudinal study of nightmares in adolescents (Nielsen et al., 2000) showing a sex difference in both DRF and nightmare recall frequency between ages 13 and 16 suggests that age-related changes in DRF manifest differently for the two sexes.

The goal of the present study was thus to examine age and sex differences in DRF in a large cohort of participants who had completed an online questionnaire about their dreams. Two different measures were available; one, a retrospective estimate of the number of dreams recalled in a typical month; the other, a dream theme diversity (DTD) score presumably assessing the lifetime prevalence of a number of different typical dream themes (Nielsen et al., 2003). As a relatively new measure, the DTD has no known correlates and its interpretation remains an open question. However, since it assesses degree of recall for all of the themes on the Typical Dreams Questionnaire (TDQ), the DTD is thought to reflect the breadth of an individual’s recall of various typical dream themes, i.e., their ability to recall a wide variety of their most common lifetime dream experiences. In this respect, the DTD can be viewed as assessing a form of episodic or autobiographical memory.

Based on the literature reviewed above, we expected to see DRF scores decrease with advancing age but especially in early adulthood. In contrast, we expected to see scores on the DTD measure increase with age, since this is a lifetime prevalence index that should increase as older participants accumulate more opportunities for recalling typical dream themes. Finally, we expected to see: age-related changes for both sexes on the DRF and DTD measures, sex differences (favoring women) on both measures, and different patterns of age-related change for the two sexes.

## Materials and Methods

### Participant sample

Records were taken from participants who had completed a 56-item version of the TDQ (Nielsen et al., 2003) and demographic items available on the “participate” portion of the *Dream & Nightmare Laboratory* website (http://dreamscience.ca/virtualdreamlab/) between January 1997 and June 2008. Participants were first informed that results from the study would be used in research and published in group form at a later date.

The data set was initially screened by an assistant who used server-logged dates, times, and IP addresses to identify and remove duplicate and spoiled records. This resulted in an initial data set containing 33,015 records. From these, 2305 records were dropped because either age was not reported (*n* = 2186) or was indicated to be <10 (*n* = 77) or >79 (*n* = 21), or excessively high estimates of dream recall (>124/month) or nightmare recall (>94/month) were given (*n* = 21). An additional 1822 records that did not contain valid scores for the retrospective monthly dream recall measure (although they did have valid DTD scores) were dropped because these subjects did not complete the entire questionnaire correctly. Thus, the sample for final analysis consisted of 28,888 participants (5884 male, 23,004 female) who possessed valid scores for the age, sex, DRF, and DTD measures. A breakdown of the cohort by Sex and Age stratum appears in Table 1. Native language was specified by 99.0% (28,608) of the sample. Of these, 82.9% (23,724) indicated their native language to be English, 13.6% indicated French (3898), and 3.4% indicated other (986). A total of 97.6% of the participants (28,198) specified an occupation; of these 50.7% indicated student (14,299), 42.5% indicated non-student (11,998), 0.9% indicated unemployed (245), and the remainder indicated specific types of employment (5.9% or 1656).

**Table 1:** **Dream recall frequency (DRF) scores by sex and age stratum**.

Sex	Age	Mean	SD	*N*
Females	10–19	1.024	0.349	8742
	20–29	1.062	0.350	7958
	30–39	1.058	0.368	3576
	40–49	1.023	0.388	1992
	50–59	0.960	0.397	612
	60–79	1.000	0.469	124
	Total	1.040	0.359	23,004
Males	10–19	0.944	0.377	2173
	20–29	0.970	0.381	2014
	30–39	0.953	0.397	894
	40–49	0.928	0.413	501
	50–59	0.973	0.453	238
	60–79	0.921	0.414	64
	Total	0.953	0.389	5884
Combined	10–19	1.008	0.356	10,915
	20–29	1.044	0.358	9972
	30–39	1.037	0.377	4470
	40–49	1.004	0.395	2493
	50–59	0.964	0.413	850
	60–79	0.973	0.451	188
	Total	1.023	0.367	28,888

Although the mean ages of males (*M* = 25.5 ± 10.6) and females (25.0 ± 10.1) differed only by 6 months, this difference was significant for such a large sample (*t*_30489_ = 2.95, *p* = 0.005). However, there were no significant age differences between the two sexes for any of the six 10-year age strata (10–19, 20–29, 30–39, 40–49, 50–59, 60–79) considered separately (all *p* > 0.185). Age (in years) was used as a covariate for some analyses.

### Dream theme diversity measure

Participants were instructed to complete the TDQ, which consists of a list of 56 themes judged in previous research to be relatively typical in the dream content of the general population (Griffith et al., 1958; Nielsen et al., 2003). The first online version of the TDQ, accounting for 84.7% of the current sample, prompted participants as follows: *For the following items, please check all of the boxes [] that apply. Have you ever dreamed of*…*?* followed by a list of 56 themes with check boxes. Each endorsed item was scored 1, otherwise 0. The second online version (see Typical Dreams Questionnaire in Appendix), accounting for 15.3% of the current sample, was modified to assess the frequency of each theme. The prompt read *Please rate how often in your life you have dreamed about each of the following themes; for each theme please circle a number from 0 to 4 as defined by this scale: 0: never; 1* = *1 time; 2* = *2*–*3 times; 3* = *4*–*10 times; 4* = *11*+ *times*. To combine the two sets of results, responses to the second version of the TDQ were recoded into a dichotomous scale equivalent to that of the first (i.e., *0* = *never*; *1* = *1 to 11*+ *times)*. The DTD total score was calculated as the number of items (out of 56) with a score of 1; the range of the scale was thus 0–56.

The TDQ, which contains the DTD themes, has been translated into several languages and the rank order of the themes found to be highly similar across cultures (Schredl et al., 2004; e.g., Yu, 2008). The DTD measure is identical to the *Divers 55* subscale of our previous study of Canadian university students (Nielsen et al., 2003), but with the addition of a single item (*56*. *encountering a kind of evil force or demon*). The *Divers 55* measure was found to have a mean score of 16.4 ± 8.2 (mode: 13; median: 15), to not differ by sex or geographic regions, and to be relatively normally distributed (Nielsen et al., 2003). A reduced, 55-item, version of the DTD was calculated in the present study for comparison with the *Divers 55* score.

### Dream recall frequency measure

Following completion of the TDQ, participants were asked to retrospectively estimate their monthly dream recall with the following question: *How many dreams of any kind do you recall in a typical month?* followed by a free response box. These responses were initially screened by a human scorer to remove all unusable responses (e.g., “lots,” “not very many,” etc.). The raw per month estimates were subsequently log-transformed [log(raw + 1)] to minimize the effect of extreme scores. This transformed variable constituted the DRF measure for final analysis.

The item on dream recall was followed by the question: *How many nightmares do you recall in a typical month?* These results have been reported elsewhere (Nielsen et al., 2006; Nielsen, 2010) and the log nightmare measure was used in the present analysis only as a covariate. The dream and nightmare recall items were followed by additional questions that varied in nature for the first and second online versions of the questionnaire and that are not assessed further here.

## Results

### Dream recall frequency

**Figure 1:**
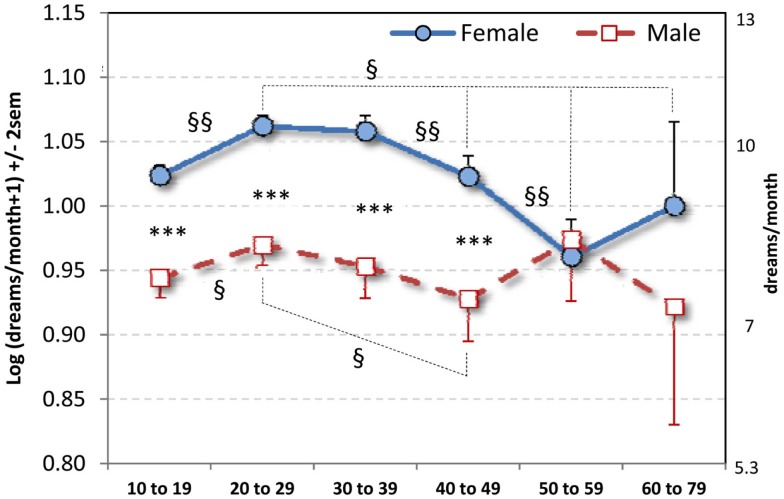
**Mean log dream recall per month (±2 SEM) for six age strata**. Equivalent number of dreams per month raw values appear on right vertical axis. *** *p* < 0.000001: comparisons between sexes; ^§§^*p* < 0.006, ^§^*p* < 0.05: comparisons between Age strata within Sex. By year, the Sex difference is apparent only for ages 14–44 (see text).

A 2 × 6 ANOVA with Sex and Age (10–19, 20–29, 30–39, 40–49, 50–59, 60–79) as independent variables and DRF as dependent variable revealed a significant Sex main effect (*F*_1,28876_ = 41.95, *p* < 0.0000001): females recalled more dreams per month than did males (Table 1). It also revealed an Age effect (*F*_5,28876_ = 8.09, *p* < 0.0000001) and a Sex × Age interaction (*F*_5,28876_ = 3.20, *p* = 0.007) such that the Sex difference obtained for Ages 10–19, 20–29, 30–39, and 40–49 (all *p* < 0.000001) but not for Ages 50–59 and 60–79 (Figure 1).

For females, contrasts revealed differences between Ages 20–29, 30–39, and all others (all *p* < 0.006) with the exception of a smaller difference between 20–29 and 60–79 (*p* = 0.05) and a marginal difference between 30–39 and 60–79 (*p* = 0.076). Age 50–59 was lower than all other ages (*p* < 0.05) except 60–79, from which it did not differ. *T*-tests conducted year-by-year for the two youngest Age strata revealed that the increase in DRF occurred at two points; first, a gradual increase from age 10 to 15 (*p* < 0.023) and, second, a relatively abrupt increase from age 18 to 20 (*p* < 0.019).

For males, contrasts revealed a significant differences between Ages 10–19 and 20–29 (*p* = 0.03) and between Ages 20–29 and 40–49 (*p* = 0.03). Year-by-year *t*-tests revealed an abrupt DRF increase between 17 and 20 (*p* < 0.023) similar to that for females.

To control for the effect of Age on the Sex difference, a univariate ANOVA with Sex as independent variable and age (in years) as a covariate was calculated. The Sex effect was still obtained (*F*_2,28887_) = 133.36, *p* < 0.0000001.

To further control for significant Sex and Age effects for nightmares found in our previous study using this cohort (Nielsen et al., 2006), the previous 2 × 6 ANOVA was repeated with an ANCOVA design using log (nightmares/month + 1) as a covariate. All three effects were still observed: Sex main effect (*F*_1,28045_ = 17.95, *p* < 0.00003), Age main effect (*F*_1,28045_ = 6.35, *p* < 0.000008), and Sex × Age interaction (*F*_5,28045_ = 2.81, *p* = 0.015).

*T*-tests conducted year-by-year on the 10–19 Age stratum revealed that the Sex effect first appeared at age 14 (*t*_885_ = −2.41, *p* = 0.017); all comparisons for prior years were not significant (all *p* > 0.20). Year-by-year *t*-tests for the 40–49 Age stratum revealed that the Sex effect disappeared definitively at age 45 (*t*_254_ = −0.44, *p* = 0.649); prior years in that stratum revealed either significant differences (all *p* < 0.02) or trends (all *p* < 0.08). Thus, a more precise estimate of the duration of the Sex difference in this sample is from ages 14–44.

There were no significant Pearson correlations between age (in years) and DRF for the entire sample (*r*_28888_ = −0.010, *p* = 0.103) or for either males (*r*_5884_ = −0.006, *p* = 0.654) or females (*r*_23004_ = −0.008, *p* = 0.246). There were also no significant Pearson correlations between age (in years) and DRF at any Age stratum for either males (all *r* > −0.052 or <0.110) or females (all *r* > −0.038 or <0.007).

### Dream theme diversity

**Figure 2:**
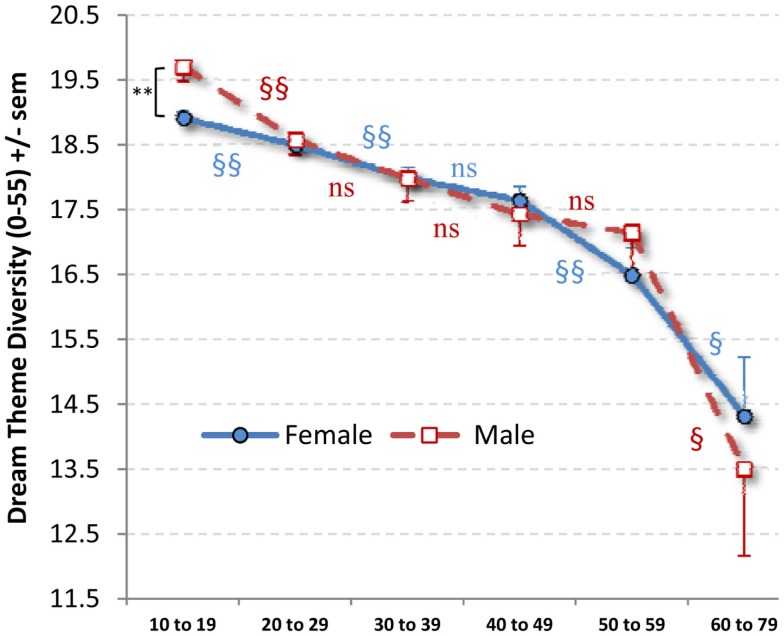
**Mean (SEM) dream theme diversity scores by Age stratum and sex**. A Sex difference was observed only for 10–19 (** *p* = 0.003). Age differences between adjacent strata within Sex (^§^*p* < 0.05; ^§§^*p* < 0.02) are indicated with red markers for males and blue markers for females.

A 2 × 6 ANOVA with Sex and Age (10–19, 20–29, 30–39, 40–49, 50–59, 60–79) as independent variables and DTD as dependent variable revealed a significant main effect for Age (*F*_5,28876_ = 22.62, *p* < 0.0000001) but no Sex main effect or Sex × Age interaction (Figure 2). The Age main effect was described best by a linear polynomial (*p* < 0.0000001) and to a lesser extent by quadratic (*p* = 0.008) and cubic (*p* = 0.011) trends.

For females, the Age main effect (*F*_5,23003_ = 16.17, *p* < 0.0000001) and linear (*p* < 0.0000001) and quadratic (*p* = 017) trends were observed. The decrease by Age stratum was significant for 10–19 to 20–29 (*p* = 0.010), 20–29 to 30–39 (*p* = 0.013), 40–49 to 50–59 (*p* = 0.014) and 50–59 to 60–79 (*p* = 0.030) but not for 30–39 to 40–49 (*p* = 0.221). The youngest and oldest strata differed substantially (*p* < 0.0000001).

For males, the Age effect (*F*_5,5883_ = 9.22, *p* < 0.0000001) and linear (*p* = 0.000002) and cubic (*p* = 0.042) trends were observed. For adjacent Age strata, the decrease was significant only for 10–19 to 20–29 (*p* = 0.001) and 50–59 to 60–79 (*p* = 0.021). The youngest and oldest strata differed substantially (*p* < 0.0000001).

Sex differences in DTD were found only at Age 10–19 (*t*_10913_ = −9.401, *p* = 0.003; all other *p* > 0.441), with males scoring higher than females. This effect was even stronger when age (in years) within the stratum was controlled as a covariate (*p* < 0.000001).

To control for the possible confounding effect of nightmares, the previous 2 × 6 ANOVA was reproduced in an ANCOVA using log (nightmares/month + 1) as a covariate. The Age main effect was still observed (*F*_5,28045_ = 11.14, *p* < 0.0000001) as was the absence of a Sex × Age interaction; moreover, the Sex main effect was now significant (*F*_1,28045_ = 4.50, *p* = 0.034). The Age main effect was again described best by a linear polynomial (*p* < 0.0000001) and to a lesser extent by quadratic (*p* = 0.023) and cubic (*p* = 0.002) trends.

Pearson correlations between age and DTD calculated separately by Sex and Age stratum revealed uniformly low coefficients. For females, the coefficients were uniformly negative and significant for 10–19 (*r* = −0.035, *p* = 0.001), 20–29 (*r* = −0.024, *p* = 0.028), and 30–39 (*r* = −0.041, *p* = 0.012), but not for 40–49 (*r* = −0.025, *p* = 0.249), 50–59 (*r* = −0.013, *p* = 0.730) or 60–79 (*r* = −0.156, *p* = 0.070). For males, the coefficients were significant for 10–19 (*r* = −0.062, *p* = 0.022), but no others (*r* = −0.036 to 0.048, all ns).

Pearson correlations revealed low, but highly significant, positive correlations between DRF and DTD for the entire sample (*r*_28888_ = 0.282; *p* < 0.0000001) and for males (*r*_5884_ = 0.276; *p* < 0.0000001) and females (*r*_23004_ = 0.287; *p* < 0.0000001) considered separately. For females, positive correlations between DRF and DTD of the same magnitude were found for every age stratum (all *p* < 0.0000001 except 60–79: *p* < 0.001); for males, positive correlations of the same magnitude were also found for every age stratum (all *p* < 0.00004) except 50–59 (*r* = 0.097, *p* = 0.134).

### Participant self-selection bias

To determine if participants visiting our (dream-themed) website were more likely than the general population to possess a higher level of dream recall, mean scores for the 55-item version of the DTD were compared with those from the identical *Divers 55* scale from our previous study of 1181 first-year Canadian University students (Nielsen et al., 2003). The latter were given the opportunity to participate in the research protocol for course credit but were nonetheless not required to do so. Participants in the previous study were aged 19.8 ± 3.9 years (males: 20.1 ± 3.60; females: 19.7 ± 3.97) and were thus compared with participants in the 10–19 and 20–29 age strata of the present study. Overall, the mean DTD score of the University student sample (16.4 ± 8.14) was about 2 points lower than that of the 10–19 age stratum (18.5 ± 10.46) and 1.5 points lower than that of the 20–29 age stratum (17.9 ± 10.02) of the present sample. For females, this value (16.4 ± 7.77) was 2.0 and 1.6 points lower for the two age strata (18.4 ± 10.23 and 18.0 ± 9.88 respectively) while for males, this value (16.3 ± 8.99) was 2.5 and 1.5 points lower (18.8 ± 11.33 and 17.8 ± 10.56). Thus, there was evidence of only a slight selection bias among both the male and female visitors to our website.

### Possible “checklist fatigue” explanation for DTD findings

**Figure 3:**
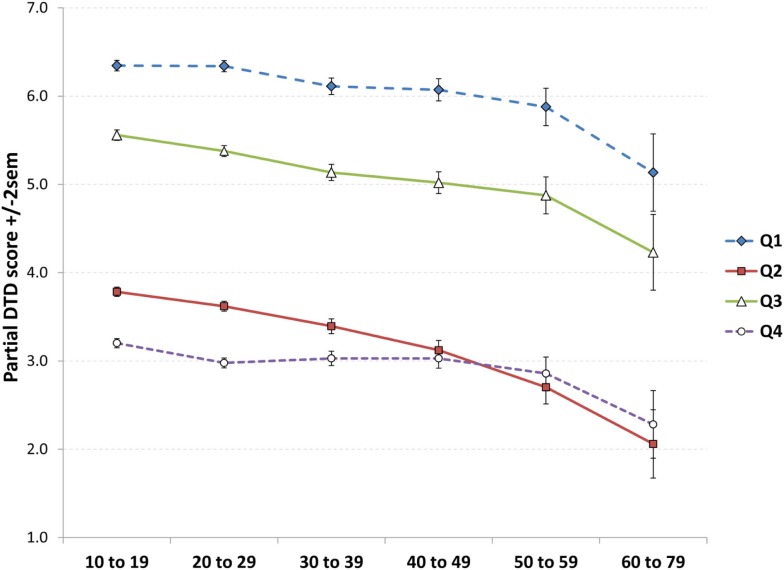
**Test of the “checklist fatigue” explanation of decreasing DTD scores with increasing age**. Scores decrease with age for all four quarters of the questionnaire, but especially Q1 to Q3, observations not consistent with the fatigue hypothesis. Similarly, the consistent occurrence of higher scores for Q3 than Q2 suggests that participants did not grow weary of filling out the checklist by Q3.

A specific pattern of results might be expected if the observed decrease in DTD scores was due to a tendency for older participants to prematurely grow weary of filling out the 56-item TDQ, i.e., progressively fewer responses with increasing age for late, relative to early, parts of the questionnaire. If not, we would expect similar age-related decreases on all parts of the questionnaire. To test this possibility, the DTD score was divided into four quarters: Q1 (items 1–14), Q2 (items 15–28), Q3 (items 29–42), and Q4 (items 43–56). The relative frequencies of these scores were then compared over age strata. A 4 × 6 ANOVA with DTD quarter (Q1, Q2, Q3, Q4) as a repeated measure and Age as an independent measure revealed a main effect for DTD quarter (*F*_3,30484_ = 7887.7, *p* < 0.0000001) such that fewer responses were given for all of the quarters with increasing age (Figure 3). Further, the highest scores were given for Q1, followed by Q3, followed by Q2 then Q4. Considered separately, each quarter showed a linear decrease with age; however, these were more robust for Q1, Q2, and Q3 (all *p* < 0.0000001) than for Q4 (*p* = 0.002) which was, in fact, better described by a cubic trend (*p* = 0.005). *Post hoc* *t*-tests for Q4 indicated an abrupt drop-off after age 10–19 (all contrasts *p* < 0.005; all strata from 20–29 to 50–59 did not differ from each other). By contrast, Q1, Q2, and Q3 all showed relatively smooth linear decreases.

## Discussion

### Decrease in dream recall frequency

Our results replicate findings from several previous cross-sectional studies showing decreased DRF with advancing age (for reviews see Funkhouser et al., 1999; Guenole et al., 2010). They are especially consistent with the finding that this DRF decrease occurs in early-to-middle adulthood rather than in later years (e.g., Herman and Shows, 1983; Giambra et al., 1996). We also demonstrate that this DRF decrease is preceded by a significant *increase* during adolescence, i.e., during the transition from ages 10–19 to 20–29. This increase replicates our previous findings in a longitudinal cohort of a DRF increase between ages 13 and 16 among both girls and boys (Nielsen et al., 2000). It also replicates findings from several other home and laboratory studies (Kales et al., 1968; Foulkes, 1982; Schredl, 2009). On the other hand, the year-by-year analyses of our youngest age groups confirmed an early DRF increase (from 10 to 15 years) among girls but not boys and a later increase (from 17 to 18 up to age 20) for both sexes. In the present study, the 10–19 and 20–29 age strata were the two largest cohorts (*N* = 10,915 and 9972) so the increases observed for these time periods are likely robust.

The increase and subsequent decrease in DRF with age were observed for both male and female participants, but sex differences in the specific patterns of change suggest that females increase access to their dreams at a younger age, maintain it at a higher level, and decrease it later in the lifespan than do males. First, both sexes show the DRF increase from 10–19 to 20–29 but, considered year-by-year, girls increase gradually between ages 10 and 15, whereas boys do not. Second, DRF for both sexes increases in the late teens (males starting at 17, females at 18) to the age of 20. Third, females maintain a DRF advantage over boys until age 44. Fourth, males start their age-related DRF decrease earlier (30–39) than do females (40–49) and also reach their DRF nadir earlier (40–49) than do females (50–59). In this respect, the findings are remarkably consistent with those of Giambra et al. (1996, see Figure 1) who found that males show an abrupt DRF decrease starting in their 30s and reaching a nadir in their 40s; women begin a slower decrease in their 30s that continues to their 50s. Our results differ slightly from these in that females in the Giambra study showed a more gradual decrease than did the males whereas the reverse was true for our findings.

In sum, different age-related patterns of DRF for the two sexes suggest an overall greater access to dreaming during much of the lifespan for females than for males. The frequency of dream recall increases for females earlier (ages 10–15), remains elevated longer (14–44), decreases more abruptly, and reaches a nadir later (50–59) than it does for males (ages 17–20, 20–29, 40–49 respectively).

Mechanisms responsible for the age-related decline in DRF observed here and elsewhere remain uncertain. An obvious possibility is the decrease in amount of REM sleep that occurs with age (see meta-analyses in Ohayon et al., 2004; Floyd et al., 2007). The most vivid dreaming is reliably recalled from REM sleep and a decrease in the latter might be expected to result in less dream recall overall. However, the age-related decline in %REM sleep is small at best, estimated to be only 0.6% per decade (2.9% total from age 19 to 75); the correlation between age and %REM is only *r* = −0.168 (Floyd et al., 2007).

Physiological features of REM sleep have also been evoked to explain the age-related decrease in dream recall. Changes in specific EEG frequencies and topographic distributions of the EEG have been linked to age-related differences in DRF (Chellappa et al., 2012), but these associations are much more apparent for NREM than for REM sleep. The phase advance and decrease in amplitude of endogenous circadian rhythms (e.g., temperature, melatonin, cortisol) with advancing age have also been proposed to influence DRF; changes in the circadian rhythm of REM sleep propensity are especially pertinent (Chellappa et al., 2009). The latter group’s use of a 40-h 75/150 min multiple nap schedule revealed that older subjects’ deficit in dream recall relative to that of young controls occurred during the subjective day, close to the peak of REM sleep propensity – the amplitude of which was also diminished in the older group. Cortisol has also been discussed as a potential marker of dream content due to its circadian patterning (Nielsen, 2004; Payne and Nadel, 2004) and its relationship to memory consolidation (Payne, 2010); changes in cortisol rhythmicity with age (increased nadir, curve flattening; Ferrari et al., 2001) may well negatively affect dream recall. Such changes are, in fact, associated with other cognitive deficits (Ferrari et al., 2001).

Other possible modulators of an age-related decrease in DRF include a diminished interest in dreams or a relative lack of current concerns that might influence dreams (Strunz, 1993), or a progressive deterioration of memory or cognitive skills such as those related to both spatial perception and dreaming (Zanasi et al., 2005). One possible cognitive mechanism, an age-related decrease in episodic and autobiographical memory, will be discussed later relative to DTD.

In sum, there is some evidence that the age-related decrease in dream recall may be influenced by a parallel decrease in %REM sleep, by changes in sleep physiology such as an advance or diminishment of the circadian rhythm of REM sleep propensity, by changes in the circadian pattern of cortisol production, or by progressive deficits in other memory and cognitive mechanisms. None of this evidence is conclusive, however, and none readily explains our observation that DRF decreases in two distinct age-related patterns for males and females.

### Sex difference

The present findings replicate a sex difference in DRF favoring females that has been observed in children, adolescents, and adults (see meta-analysis in Schredl and Reinhard, 2008). Because of our large sample, we were able to determine with some confidence that this sex difference appears in early adolescence (age 14) and (with less confidence) that it disappears in middle adulthood (age 44). The precision of these findings may help explain why some previous studies have identified a sex difference in DRF for children, i.e., because they grouped participants who were both younger and older than age 14.

Although there is no definitive explanation for the sex difference reported here, the cognitive processes of women are known to differ from those of men in a number of basic ways that might be related to superior recall of dream content. These include enhanced processing of novel visual stimuli (Yuan et al., 2012) and facial expressions (McClure, 2000), superior performance on several social cognition tasks (Gur et al., 2012), and better memory for emotional stimuli (Canli et al., 2002), episodic stimuli (Herlitz and Rehmann, 2008), and autobiographical episodes (Seidlitz and Diener, 1998; Fivush, 2011; Zucco et al., 2012), whether such episodes are positive, negative, or neutral in affective tone (Seidlitz and Diener, 1998). The neurophysiological differences contributing to sex differences in emotional processing are well-known (Cahill et al., 2004; Andreano and Cahill, 2009), including those underlying sex differences in autobiographical memory (Piefke and Fink, 2005). As there is good evidence that episodic and autobiographical memory processes also diminish with age, this may be the most appropriate explanation for the observed sex difference. In short, superior dream recall may be but one expression of an underlying advantage that women enjoy in the processing and remembering of novel, emotional, social imagery.

### Dream theme diversity findings

Findings for the DTD measure contradicted our expectation that the prevalence of typical dream themes would accumulate with increasing age. Rather, DTD scores decreased monotonically with age for both sexes. This unexpected finding forces us to reconsider our initial interpretation of the DTD measure in light of several alternatives. A first possibility that we examined in our analyses considered that DTD scores may have decreased with age because of “checklist fatigue” or the possible tendency for a subject’s motivation to complete the entire 56-item questionnaire to wane with age. We found evidence that this was likely not the case in that linear decreases with age were seen for all four quarters of the questionnaire when considered separately and that such a decrease was *least* evident for the fourth quarter of the questionnaire where the steepest decrease would have been expected. A related consideration is that instructions for completing the questionnaire may not have been properly understood to refer to dreams recalled over the *entire* lifespan and participants therefore only reported dreams they could recall from recent memory. While it seems unlikely that all participants would misconstrue the instructions in this manner, the possibility would imply that the DTD findings are due to an age-related decline in recall for recent typical dreams.

A second possible explanation of the DTD findings is that the typical dreams comprising this measure are sensitive to generational differences in life experiences. Older participants may have had fewer of the life experiences that are thought by some (e.g., Freud, 1900) to trigger typical themes such as those on the questionnaire. By this reasoning, for example, fewer experiences with airplane flight by older individuals might be reflected in a lower likelihood of having flying dreams, or less witnessing of violence might lead to a lower likelihood of having attack dreams, and so forth. While this possibility could not be addressed directly with the present measures, evidence for such an effect was found to partially explain a progressive age-related decrease in color dreams (Schwitzgebel, 2003; Murzyn, 2008), specifically, older subjects had both fewer color dreams and more past exposure to black and white televisions (Okada et al., 2011). Notwithstanding this finding, the latter authors also found that the effect size of the generational influence on color dreams was only one twentieth of the effect size observed for aging *per se*. The generational explanation is also not consistent with the fact that Griffith et al.’s (1958) early cohorts of American and Japanese participants had a mean diversity proportion of 44% (calculated out of a total of 34 typical dream items) which was much higher (not lower) than those in the present study for any Age stratum, which were (from youngest to oldest) 35, 33, 32, 31, 31, and 24% (calculated out of 56 items). Future studies could further assess this hypothesis by examining age-related changes in individual DTD items for which associated life experiences have either remained constant over generations (e.g., losing teeth, falling down) or have become less, not more, accessible to more recent generations (e.g., seeing snakes, seeing violent wild beasts).

A third possible explanation is that the DTD decrease observed here reflects a parallel age-related decrease in nightmare frequency reported elsewhere. For a participant cohort largely overlapping with the present one we previously showed a decrease for the nightmare frequency measure, log (nightmares + 1), that had Sex, Age, and Sex × Age interactions that were similar to the ones for DRF found in the present study (see Figure 1 in Nielsen et al., 2006). However, this possibility is not supported by the fact that, when the log nightmare measure was controlled as a covariate in the present analyses, the main effects and interactions for both DRF and DTD measures were maintained.

A final possibility – and one favored here – is that the DTD is effectively a measure of one form of episodic or voluntary autobiographical memory; accordingly, its age-related decrease may be an expression of similar decreases that have been demonstrated for these latter memory systems (Piolino et al., 2002). Many studies indicate that both episodic memory and autobiographical memory (i.e., memory for episodes that are personally significant and emotional in nature) are reduced with age whereas semantic memory is preserved (Kensinger, 2009; St-Laurent et al., 2011). In fact, older adults, relative to younger adults, show a decrease in the episodic richness of autobiographical memories, i.e., in the proportion of specific episodic details relative to semantic information (Levine et al., 2002; Piolino et al., 2002; St Jacques and Levine, 2007). This decrement is apparent for autobiographical memories that are voluntarily evoked in response to cues (such as for the items of the TDQ), but not for autobiographical memories that occur involuntarily (Schlagman et al., 2009).

To illustrate these findings, one study (Levine et al., 2002) using a standardized *Autobiographical Interview* method, demonstrated that younger adults who were asked to recall memories from five distinct life periods reported mainly episodic details reflecting happenings, locations, perceptions, and thoughts whereas older adults reported primarily semantic details not connected to a particular time and place. This group difference persisted even when subjects were specifically probed to report contextual details. Thus, the DTD in the present study may be a type of cued-recall autobiographical memory measure analogous to the *Autobiographical Interview* in that it probes for the simple recognition of 56 high-probability dream themes also occurring in different life periods. And, like other cued-recall measures, the DTD may be sensitive to age-related memory declines.

An exception to the conclusion that the richness of autobiographical memories is attenuated with age is the fact that many such memories tend to date from adolescence/early adulthood, a phenomenon referred to as the “reminiscence bump” (Schlagman et al., 2009). Memory associations to dreams have, in fact, been found to conform to this curvilinear reminiscence pattern for older but not younger subjects (Grenier et al., 2005). The existence of the reminiscence bump is consistent with the notion that recall for some long-past autobiographical material remains as good as, or even better than, it is for more recent material. Such a disproportionate memory for remote material is referred to by some as a “temporal gradient” and has been found repeatedly in pathological amnesias such as Korsakov’s syndrome but also less severely in normal aging using past media-related events as probes (Bizzozero et al., 2008). Thus, to the extent that typical dream themes originate in adolescence/early adulthood, evidence of an age-related autobiographical memory decline may not be a sufficient explanation for the observed DTD results. Rather, the results may reflect some other type of cognitive or affective deficit, such as memory reconsolidation. On the other hand, it is not known whether most TDQ themes in fact do date from the adolescence/early adulthood time period. Moreover, that the reminiscence bump occurs for important and positive memories but not sad, traumatic, or negative memories (Berntsen and Rubin, 2002; Thomsen et al., 2011) suggests that positive DTD items would be less likely than negative items to suffer age-related decreases. Given that many of the DTD items are highly emotional in nature in either a positive (e.g., flying, sexual experiences, finding money) or a negative (e.g., being chased, being attacked, being tied up, dying) sense, they may well be differentially sensitive to the autobiographical reminiscence bump. This alternative explanation of the DTD findings could be tested further by determining if age-related decreases occur for individual DTD items whose emotions are highly positive vs. negative in nature.

To summarize, a number of alternatives may be considered in explaining the unexpected age-related decrease in dream diversity observed here. Some of these, such as age-related changes in REM sleep physiology and circadian regulation or generational differences in opportunities for life experience, are supported by only a limited number of studies whose effects are small. A more parsimonious explanation may be that the DTD measure reflects age-related changes in episodic and autobiographical memory that have been demonstrated in a number of other contexts. If so, this interpretation raises questions about how participants come to forget typical dreams that they may once have experienced and remembered, which types of typical dreams are more likely to be forgotten in this manner, and whether the reliable age-related decreases in DRF may, too, be partly explained by such changes in episodic and autobiographical memory.

### Validity of internet samples

It is now widely accepted that Internet samples constitute a valid source of information about self-reported human behavior; such samples may even be superior to other sampling methodologies in several respects. The use of Internet surveys has increased markedly in many areas, including sleep medicine (Mindell et al., 2010; e.g., Saxvig et al., 2012) and dreaming (e.g., Cheyne et al., 1999; Brand et al., 2011) as well as in other areas that concern sensitive personal information. Surveys of sexual health (e.g., Foster et al., 2010) and the use of illicit drugs (e.g., Noack et al., 2011) are but two of many examples. Advantages of Internet surveys include the sharing of information and experiences that might not be disclosed by other means, reduction of social desirability and yea-saying biases, reduction of error, and access to hidden and hard-to-reach populations (for review see Rhodes et al., 2003). Online surveys have been validated against paper-and-pencil tests (Knapp et al., 2004), mail surveys (McCabe, 2004), and national population studies (Ross et al., 2005), with high concordances between methods having been reported (Mindell et al., 2010; e.g., Saxvig et al., 2012).

In contrast, health-oriented web sites such as our own may attract more individuals who suffer from health difficulties; our site is particularly likely to attract individuals with an interest in sleep, dreaming, and nightmares. Since females are more likely than males to seek help, especially for emotional problems (Moller-Leimkuhler, 2002), a self-selection gender bias may have influenced the composition of our sample. There may also be self-selection biases toward younger, more educated and more affluent respondents, such as those who can afford Internet access (Ross et al., 2005; Mindell et al., 2010). Additionally, the sample of the present study may have been biased in that responses by participants who did not respond to DRF items with quantifiable numbers were excluded.

## Conflict of Interest Statement

The author declares that the research was conducted in the absence of any commercial or financial relationships that could be construed as a potential conflict of interest.

## Appendix

### Typical dreams questionnaire

Please rate how often in your life you have dreamed about each of the following themes; for each theme please circle a number from 0 to 4 as defined by this scale:

**Table:**

**0, never; 1, 1 time; 2, 2–3 times; 3, 4–10 times; 4, 11+ times**.						
1	Being chased or pursued, but not physically injured	0	1	2	3	4
2	Being physically attacked (beaten, stabbed, raped, etc.)	0	1	2	3	4
3	Trying again and again to do something	0	1	2	3	4
4	Being frozen with fright	0	1	2	3	4
5	Eating delicious foods	0	1	2	3	4
6	Arriving too late, e.g., missing a train	0	1	2	3	4
7	Swimming	0	1	2	3	4
8	Being locked up	0	1	2	3	4
9	Snakes	0	1	2	3	4
10	Finding money	0	1	2	3	4
11	Flying or soaring through the air	0	1	2	3	4
12	Falling	0	1	2	3	4
13	Being inappropriately dressed	0	1	2	3	4
14	Being nude	0	1	2	3	4
15	Being tied, unable to move	0	1	2	3	4
16	Having superior knowledge or mental ability	0	1	2	3	4
17	Creatures, part animal, part human	0	1	2	3	4
18	Your teeth falling out/losing your teeth	0	1	2	3	4
19	Seeing yourself in a mirror	0	1	2	3	4
20	Having magical powers (other than flying or floating through the air)	0	1	2	3	4
21	Floods or tidal waves	0	1	2	3	4
22	Tornadoes or strong winds	0	1	2	3	4
23	Earthquakes	0	1	2	3	4
24	Insects or spiders	0	1	2	3	4
25	Being a member of the opposite sex	0	1	2	3	4
26	Being an object (e.g., tree or rock)	0	1	2	3	4
27	Being killed	0	1	2	3	4
28	Seeing yourself as dead	0	1	2	3	4
29	Vividly sensing, but not necessarily seeing or hearing, a presence in the room	0	1	2	3	4
30	Being unable to find, or embarrassed about using, a toilette	0	1	2	3	4
31	School, teachers, studying	0	1	2	3	4
32	Sexual experiences	0	1	2	3	4
33	Losing control of a vehicle	0	1	2	3	4
34	Fire	0	1	2	3	4
35	A person now dead as alive	0	1	2	3	4
36	A person now alive as dead	0	1	2	3	4
37	Being on the verge of falling	0	1	2	3	4
38	Failing an examination	0	1	2	3	4
39	Being smothered, unable to breathe	0	1	2	3	4
40	Wild, violent beasts	0	1	2	3	4
41	Being at a movie	0	1	2	3	4
42	Killing someone	0	1	2	3	4
43	Lunatics or insane people	0	1	2	3	4
44	Being half awake and paralyzed in bed	0	1	2	3	4
45	Seeing a face very close to you	0	1	2	3	4
46	Seeing a UFO	0	1	2	3	4
47	Seeing extra-terrestrials	0	1	2	3	4
48	Traveling to another planet or visiting a different part of the universe	0	1	2	3	4
49	Being an animal	0	1	2	3	4
50	Being a child again	0	1	2	3	4
51	Seeing an angel	0	1	2	3	4
52	Encountering God in some form	0	1	2	3	4
53	Discovering a new room at home	0	1	2	3	4
54	Seeing a flying object crash (e.g., airplane)	0	1	2	3	4
55	Someone having an abortion	0	1	2	3	4
56	Encountering a kind of evil force or demon	0	1	2	3	4

Other (please describe)

Which theme occurred *most often* in your life (please specify number from 1 to 56)? ________.

Which theme occurred *earliest* in your life (please specify number from 1 to 56)? ________ At what age?________years.

How many *dreams* of any kind do you recall *in an average month (circle one)*? 0, 1–2, 3–5, 6–10, 11–15, 16–20, 21–25, 26–30, 31+.

How many *nightmares* do you recall *in an average month (circle one)*? 0, 1–2, 3–5, 6–10, 11–15, 16–20, 21–25, 26–30, 31+.
